# Spring Leafing Phenology Favors Younger Culms of Moso Bamboo: Aspects From Water Use Relations

**DOI:** 10.3389/fpls.2020.00550

**Published:** 2020-05-08

**Authors:** Tingting Mei, Xiang Liu, Dongming Fang, Guomo Zhou, Chongyu Ye, Pingheng Li, Yongjun Shi, Huaqiang Du, Frank Berninger, Dirk Hölscher

**Affiliations:** ^1^State Key Laboratory of Subtropical Silviculture, Zhejiang A&F University, Lin’an, China; ^2^Key Laboratory of Carbon Cycling in Forest Ecosystems and Carbon Sequestration of Zhejiang Province, Zhejiang A&F University, Lin’an, China; ^3^Department of Environmental and Biological Sciences, University of Eastern Finland, Joensuu, Finland; ^4^Tropical Silviculture and Forest Ecology, University of Goettingen, Göttingen, Germany

**Keywords:** age, sap flow, transpiration, water distribution, water dynamic

## Abstract

As the most widely distributed giant running bamboo species in China, Moso bamboo (*Phyllostachys edulis*) can accomplish both development of newly sprouted culms and leaf renewal of odd-year-old culms within a few months in spring. The two phenological events in spring may together change water distribution among culms in different age categories within a stand, which may differ from our conventional understanding of the negative age effect on bamboo water use. Therefore, to explore the effect of spring shooting and leaf phenology on age-specific water use of Moso bamboo and potential water redistribution, we monitored water use of four culm age categories (newly sprouted, 1-, 2-, and 3-year-old; namely A0, A1, A2, A3) in spring from March to June 2018. For newly sprouting culms, the spring phenological period was classified into five stages (incubation, culm-elongation, branch-development, leafing, established). Over these phenological stages, age-specific accumulated sap flux density showed different patterns. The oldest culms, A3, were not influenced by leaf renewal and kept nearly constant and less water use than the other aged culms. However, A2, which did not renew their leaves, had the most water use at the two initial stages (incubation, culm-elongation) but consumed less water than A0 and A1 after the fourth stage (leafing). At the end of June, water use of the four age categories sorted in order of A0 > A1 > A2 > A3, which confirms the conventional thought and observations, i.e., a negative age effect. The results indicate that new leaf flushing may benefit younger culms (A1 and A0) more than older culms (A2 and A3), i.e., increasing their transpiration response to radiation and share of the stand transpiration. With the underground connected rhizome system, the bamboo stand as an integration seems to balance its water use among culms of different ages to support the water use of freshly sprouted culms during their developing period.

## Introduction

Moso bamboo (*Phyllostachys edulis*), the most widely distributed giant running bamboo species in China (more than 4 million ha in 2005; Song et al., 2011), is high water-demand plant (Li et al., 2019; Shi et al., 2020) and inversely may impact local hydrologic cycling and regional water balance (Komatsu et al., 2010; Gu et al., 2019) and thus has been increasingly studied in recent years. Previous studies found that Moso bamboo forests can transpire 324–567 mm per year depending on stand densities in Japan (4,000 culms ha^–1^, Komatsu et al., 2010; 11,000 culms ha^–1^, Ichihashi et al., 2015) and in China (3,600 culms ha^–1^, Zhao et al., 2017b). On the culm scale, maximal culm sap flux density of Moso bamboo (∼20 g cm^–2^ h^–1^; DBH: 11 cm) is similar to neighboring trees (DBH: 13.5–44.6 cm; Kume et al., 2010; Komatsu et al., 2010) and some giant clumpy bamboo species, e.g., *Bambusa blumeana* in the Philippines (DBH: 9.9 cm; Dierick et al., 2010), *Bambusa vulgaris* in China (DBH: 9.5 cm; Yang et al., 2015), *Dendrocalamus asper* in Indonesia (DBH: 11.9 cm; Mei et al., 2016). In these previous studies, the effects of age and phenology on bamboo water use have been explored gradually in recent years (Komatsu et al., 2010; Tsuruta et al., 2016; Zhao et al., 2017a, b; Gu et al., 2019).

**FIGURE 1:**
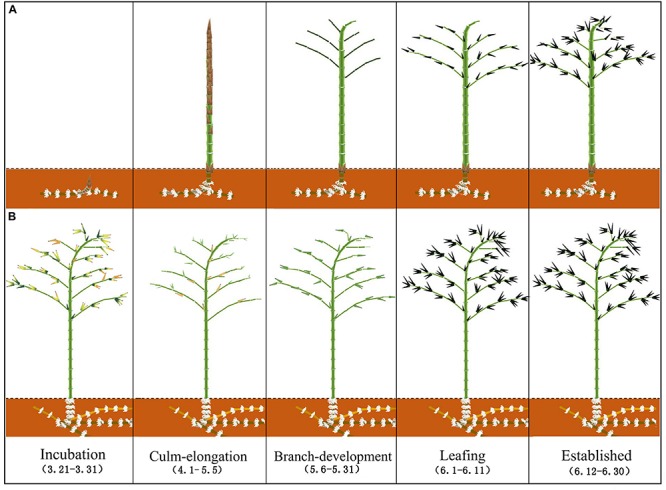
Phenological stages of **(A)** sprouting new culms and **(B)** leaf shedding and flushing on odd-year-old established culms. Incubation refers to the stage when shoots stay under the ground. In the meantime, leaves of odd-year-old culms remain on branches but turned to yellow. Culm-elongation refers to the stage when freshly sprouted culms grow above the ground and accomplish their height growth. During the stage, odd-year-old established culms shed old leaves and flush new leaves. Branch-development refers to the stage when the freshly sprouted culms accomplish branch growth, and in the same period, new leaves are expanding on the odd-year-old established culms. Leafing refers to the stage when freshly sprouted bamboo culms start flushing leaves while odd-year bamboo culms almost finish leaf expanding. Established refers to the stage when freshly sprouted bamboo culms finish their culm and leaf growth.

Moso bamboo is a monocotyledonous species, finishes culm growth within 1–2 months in its first growing season, and in the remaining life, it will not renew the vascular system by secondary growth as dicotyledonous trees do (Liese and Köhl, 2015). Water-conducting failure in the vascular system may thus increase with age due to embolism and accumulating tyloses and depositions, which may lead to decreasing water use with increasing ages (Liese and Weiner, 1996). Some studies have confirmed such negative age effects on the water use of Moso bamboo (Zhao et al., 2017b; Gu et al., 2019). However, there is also a study that indicates an insignificant age effect on water use of 1-, 2-, and more than 3-year-old culms (Tsuruta et al., 2016). This finding implies that culm water use rates may not always decline with age in Moso bamboo. Excepted the reported positive embolism-repair function from root pressure (Wang et al., 2011; Cao et al., 2012), another supposed reason for the differential age effect may be the bamboos’ ability of source integration, which enables bamboo culms connected within a grove to redistribute water among them via rhizome system (Fang et al., 2019; Mei et al., 2019). Such a redistribution characteristic is particularly important for newly sprouted culms in spring, as they are leafless before accomplishing culm and branch elongation and thus may rely on their neighboring established culms. Based on the establishing process of newly sprouted culms, the spring phenological period (March to June) of Moso bamboo was classified into five stages (incubation, culm-elongation, branch-development, leafing, established; Figure 1). Newly sprouted culms have been staying underground in incubation stage (Before Late March), sprouting from the soil and elongating in culm-elongation stage (Early April–Early May), flushing new branches in branch-development stage (Early May–end of May), flushing leaves in leafing stage (Early June–Mid June), and maintaining their fully-grown leaves in established stage (Mid June–Late June and thereafter).

During the same spring period when newly sprouted culms develop, some established culms are renewing their leaves, which may further change the water use among different aged culms in a grove. Moso bamboo renews its leaves in spring when establishing new culms (Li et al., 1998a). Freshly sprouted Moso bamboo culms (A0) in a given year will drop their leaves after 1 year (A1) and increase the leaf life span thereafter to 2 years. Such a leaf-changing rhythm means that Moso bamboos drop their leaves in odd years (A1, A3, A5, and so on) and keep the leaves in even years (A2, A4, and so on; Li et al., 1998a, b). New leaves start to flush almost immediately after old leaves are shed. Odd-year culms have been turning their foliage yellow in the incubation stage, shedding and flushing their leaves in the culm-elongation stage, expanding new leaves in the branch-development stage, and maintaining their fully grown leaves in leafing stage and established stage. Even-year culms (A2, A4, and so on) maintain their leaves on branches and do not have phenological changes. Sprouting of new culms and leaf-renewing of old culms together may impact and change water use dynamics of different aged culms, which may lead to water redistribution among different aged culms within a stand.

The coordinated leaf phenology and shoot-sprouting may change carbon and water circulation within the bamboo forest (Li et al., 1998b). In the shoot sprouting period, non-structural carbohydrates (Song et al., 2016) and nutrients (Li et al., 1998a, b) in established culms are implied to be transferred to the connected freshly sprouted culms. As a carrier of carbohydrate and other nutrients, water use of Moso bamboo in different ages may differ in spring phenological stages (pre- and post-leaf changing and shoot sprouting) due to both changing leaves and sprouting new culms as has been indicated for other, clumpy bamboo species (Fang et al., 2019; Mei et al., 2019). With leaf aging, stomatal conductance decreases generally, and leaf renewing is a way to retrieve the function (Davis and Mccree, 1978; Field and Mooney, 1983). During the time when leaf shedding and flushing, water use of those involved bamboo culms may experience a transition from less to more with shedding older leaves and flushing new leaves. Additionally, bamboo culms are connected via a rhizome system, which would allow allocating water among different age categories toward freshly sprouted culms. Shedding leaves in some culms may reduce the stand transpiration but may benefit other culms to obtain more water. However, it is still unknown how and when water use of Moso bamboo culms will change during the specific spring phenology.

In this study, sap flux densities in Moso bamboo culms with four age categories (freshly sprouted, 1-, 2-, and 3-year-old, named as A0, A1, A2, and A3) were monitored from March to June in 2018 during spring phenological stages. The study aimed at revealing the influence of spring phenology (changing leaves and shooting) on age-specific water use dynamics of different aged Moso bamboo, to deepen our insight on potential water redistribution within bamboo stands. Furtherly, the work may help us to better understand water use strategy of different aged Moso bamboo culms and their response as a whole to changing conditions, which may provide some background support for the management of bamboo forests.

## Materials and Methods

### Study Area and Bamboo Selection

The experiment was conducted in a Moso bamboo stand, which is located in the experimental garden (30°15′55"N, 119°42′47"E, 13 masl) of Zhejiang A&F University, Hangzhou, China. Hangzhou is in the subtropical monsoon climate zone. From 2008 to 2017, mean annual temperature of the study site was 17.6 ± 0.4°C and annual rainfall was 1,579.7 ± 263.5 mm (mean ± STD; data from National Meteorological Information Center^1^). During the experimental period from 22 March to 30 June 2018, the experimental site had a mean daily temperature of 21.5 ± 4.7 mm, solar radiation of 27.8 ± 12.5 mol m^–2^ day^–1^, vapor pressure deficit of 0.75 ± 0.44 kPa, soil moisture of 0.24 ± 0.02 m^3^ m^–3^, and a total rainfall of 541.6 mm.

The studied bamboo stand was established in 2007. The stand produces similar amounts of freshly sprouted bamboo culms each year, which is different from the typical biannual production cycle in many stands characterized by an alternative high or low shoot production every 2 years (Song et al., 2016; Zhou et al., 2018). The bamboo stand was intensively managed before it was used as an experimental site and the top canopies of some old bamboo culms (sprouted before 2015) were cut. However, canopies of young bamboo culms freshly grown in recent 3 years were kept and a mean height of bamboo with entire canopy is 12.22 m. The stand density is 1,767 culm ha^–1^ and means diameter at breast height (DBH) is 8.76 cm. Four age categories (freshly sprouted, 1-, 2- and 3-year-old; named as A0, A1, A2, and A3) were selected, and three to four culms per category were monitored in this study (Table 1). The selected bamboo culms had entire and unbroken crowns and were not affected by visible diseases or pests. DBH for each selected culm represents 95% of the DBH range in the bamboo stand.

**TABLE 1:** Basic information of the studied Moso bamboo and identification for culm ages.

						**Appearance characteristics for identifying ages**		
						**Culm surface**	**Sheath status (near the ground)**	**Sheath ring**
**Age**	**Sprouting year**	**Monitored number**	**DBH (cm)**	**Culm wall thickness (cm)**	**Density (culm ha^–1^)**			
A0	2018	6	10.4 (1.2)	–	450	Shiny light green; fine white powder	Intact	White bristles
A1	2017	3	11.7 (0.9)	1.2 (0.3)	500	Shiny light green; fine white powder	Intact	White bristles
A2	2016	4	8.8 (1.0)	0.9 (0.2)	625	darker green; fewer white powder	Worn a little	Yellow bristles
A3	2015	4	9.5 (0.4)	1.1 (0.0)	550	dark and white dotted; barely powder	Worn severely or disappeared	Brown or none bristles

### Age Identification

To identify the ages of the selected culms, at first, we checked records about the freshly sprouted culms from 2015 to 2017 and labeled them as the 1-, 2- and 3-year-old culms. Second, we double-checked the ages according to culm characteristics for each specific age (Zhao et al., 2017a, b). Briefly, different aging culms differ in culm color, amount of coated white powder, and wear-out extents of sheaths at culm base. Compared to older culms, younger culms usually have green surfaces covered with white powders, more intact culm sheaths near the ground, and more light-colored bristles on the sheath ring around nodes (Table 1).

### Sap Flow Measurement

Bamboo sap flow at breast height was measured with self-constructed 10-mm-length thermal dissipation probes (TDP; Granier, 1985). Three to four culms per category were monitored in this study (Table 1), and two pairs of TDP sensors were installed on each monitored culm. Each pair of TDP constitutes a heating probe and a reference probe, and the temperature differences between the probes are used for calculating stem/culm sap flux densities. In the field, the probes were first protected by 2.5 mm-diameter aluminum tubes, and then they were vertically inserted into culms with 10 cm spaces axially between the probes and the heating probe located in the upper position (Mei et al., 2016). After installation, the probes were covered with reflecting film and plastic shield to protect them from radiation and rainfall. The upper heating probe was heated with 0.1 W power and the lower reference probe was unheated. The temperature differences between the two probes were recorded as voltage signals by a data logger and a multiplexer (CR1000 and AM16/32, Campbell Inc., United States). The signals were scanned every 30 s and averaged every 10 min.

According to the principal theory of TDP, i.e., the temperature differences are negatively related with sap flux density due to the heat dissipation effect of the water (Granier, 1985; Lu et al., 2004), Granier (1985) first built up an empirical equation based on calibration with three tree species. However, it was recommended to conduct a species-specific calibration for TDP when applying on a new species (Bush et al., 2010; Steppe et al., 2010). In our study, sap flux density (g m^–2^ s^–1^) was derived with Eq. 1 specified for adult Moso bamboo (Zhao et al., 2017b) and Eq. 2 for newly sprouted bamboo (Fang et al., unpublished data).

(1) $$J_{S\,\_\,E\,S}=306.5\times {\left(\frac{V_{\max}}{V}-1\right)}^{1.746}$$

(2) $$J_{S\,\_\,N\,S}=3.99\times 119\times {\left(\frac{V_{\max}}{V}-1\right)}^{1.231}$$

where *J*_S_ES_ and *J*_S_NS_ are sap flux densities of established and newly sprouted culms, respectively; *v* is the output voltages indicating the temperature difference between the two probes of TDP, *v*_max_ is the maximal value of *v* during a day.

To further calculate the whole culm water use, a cross-sectional area (*A*_cross–section_; cm^–2^) of the studied bamboos was determined from DBH (Eq. 3).

(3) $$A_{\mathrm{cross}-\mathrm{section}}=7.219\times \text{DBH}-41.932\;\left(R^{2}=0.86;P<0.01\right)$$

Age-specific stand transpiration (only included culms sprouted in the latest 3 years, all of them have un-cut crown) was calculated with Eq. 4, and the relative share of each age-specific stand transpiration in a given date was derived by dividing each age-specific stand transpiration with the age-summarized stand transpiration.

(4) $$T_{\text{age}\,\_\,i}=\frac{\sum J_{S\,\_\,\mathrm{daily}\,\_\,\mathrm{culm}}\times A_{\mathrm{cross}\,\_\,\mathrm{area}\,\_\,\mathrm{culm}}}{n_{i}}\times N_{i}$$

where *i* is age (0, 1, 2, 3); *J*_s_daily_culm_ and *A*_cross_area_culm_ are daily accumulated sap flux density and cross-sectional area at breast height of each monitored culm; *n*_i_ and *N*_i_ are total numbers of monitored culms and stand culms of age *i*, respectively.

### Micrometeorological Factors and Soil Moisture Measurement

A micrometeorological station was set up above the bamboo canopy. The station measured photosynthetic photon flux density (LI190R, Campbell Scientific, United States), air temperature and humidity (HMP155A, Campbell Inc., United States). Soil moisture in the plot (0–30 cm depth) was measured with three probes (CS616, Campbell Inc., United States). All data were recorded with the same time steps as that of TDP sensors.

### Statistical Analysis

To explore temporal dynamics of different aged culms, we averaged daily accumulated sap flux densities (*J*_s_daily_; g cm^–2^ day^–1^) of each age category (3–6 culms used) in each day and plotted the average daily accumulated sap flux densities against time.

Further, analysis of variance (ANOVA) was conducted to detect the effect of age and phenological stage on *J*_s_daily_. With Gabriel’s test (designed for unbalanced group sizes), multiple comparisons of *J*_s_daily_ were conducted among phenological stages in a given age as well as among ages in a given phenological stage.

The response of *J*_s_daily_ to daily accumulated radiation was explored by fitting them with linear regression for each given age in a given stage. Additionally, to compare the different responses of ages, slopes of the linear regression between *J*_s_daily_ and daily accumulated radiation in each given stage were examined homogeneity among ages.

All plots and analyses were performed with SAS 9.4 (SAS Institute Inc., Cary, NC, United States).

## Results

### Age-Specific Sap Flux of Moso Bamboo Over Spring Phenological Periods

**FIGURE 2:**
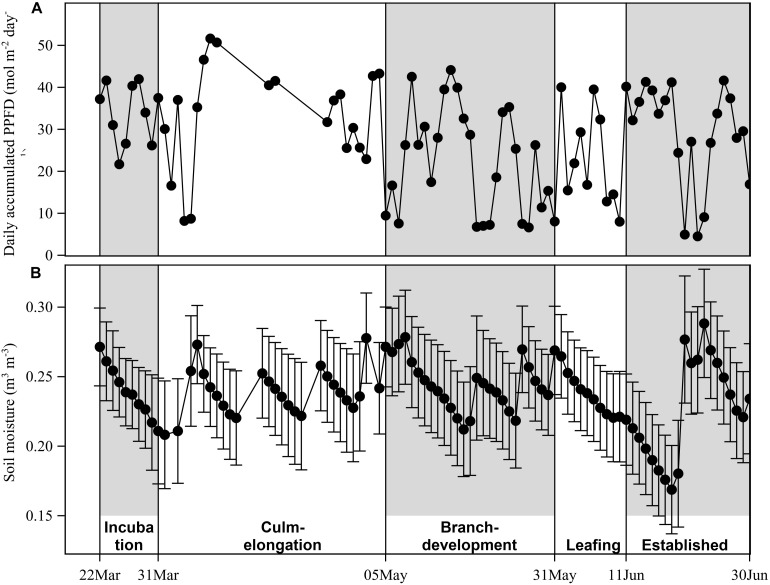
**(A)** Daily accumulated photosynthetic photon flux density (PPFD) and **(B)** daily averaged soil moisture over five phenological stages (incubation, culm-elongation, branch-development, leafing, established stages of newly sprouted culm). The presented daily averaged soil moisture are averaged values from 3 sensors in the plot, and the standard deviation of daily averaged soil moisture is presented with error bars. Daily PPFD was obtained from one sensor from a micrometeorological station, so there is no variance presented here due to lack of replicates.

**FIGURE 3:**
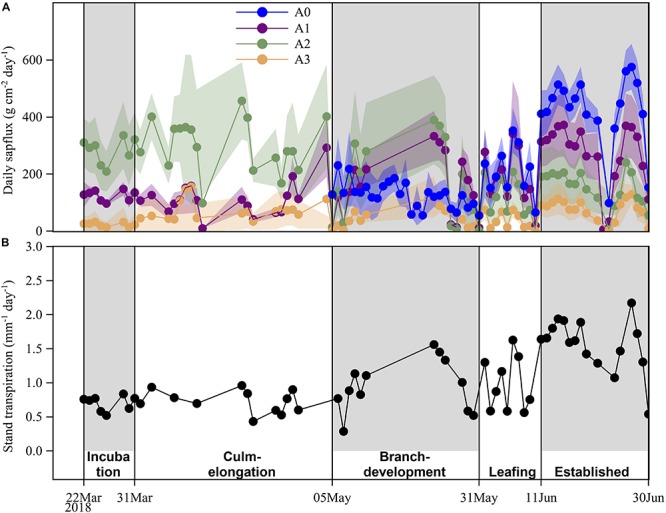
**(A)** Age-specific daily accumulated sap flux of the four age categories (**A0**, **A1**, **A2**, **A3**) and **(B)** stand transpiration of the studied Moso bamboo stand over five phenological stages (incubation, culm-elongation, branch-development, leafing, established stages of newly sprouted culm). The presented age-specific sap flux densities are averaged values from 3 to 6 bamboo culms for each age category, and the standard deviation of daily age-specific sap flux densities is presented with the corresponding color band for each age. Daily stand transpiration was obtained from one plot, so no variation is presented here due to lack of replicates.

Bamboo spring phenology was classified into five periods according to phenological events of sprouting new culms and flushing new leaves for freshly sprouted bamboos and adult bamboos in this study (Figure 1). Important phenological events included leaf flushing for odd-year-old established culms in the culm-elongation stage of A0, and leaf flushing for A0 in the leafing stage and culm development in the culm-elongation stage. Over the five spring phenological periods from 22 March to 30 June 2018, averaged daily accumulated photosynthetic photon flux density (PPFD) was 27.8 ± 12.5 mol m^–2^ day^–1^ and averaged daily mean soil moisture was 0.24 ± 0.02 m^3^ m^–3^ (Figure 2). Daily accumulated sap flux density (*J*_s_daily_, g cm^–2^ day^–1^) showed different dynamic patterns among the three adult ages, i.e., 1-, 2- and 3-year-old culms (A1, A2, and A3) showed a slow increasing, decreasing, and nearly constant overall pattern, respectively (Figure 3A). For A0, *J*_s_daily_ increased from branch-development through leafing to established stages. An obvious increase of *J*_s_daily_ was observed between culm-elongation and branch-development stages of A0 for A1, while an obvious drop of *J*_s_daily_ was observed between branch-development and leafing stages of A0 for A2 (Figure 3A).

**FIGURE 4:**
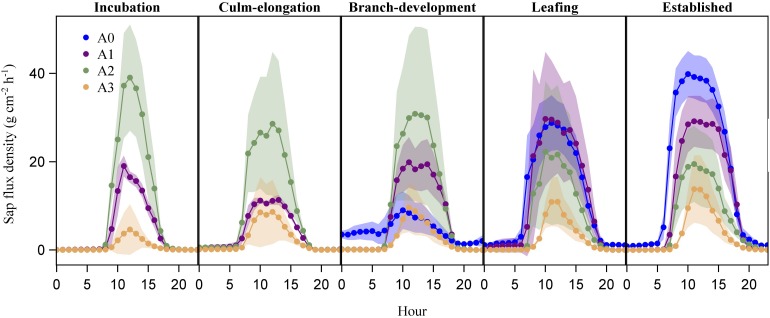
Age-specific sap flux density (mean + STD) of Moso bamboo over five spring phenological stages (incubation, culm-elongation, branch-development, leafing, established stages of newly sprouted culm). The presented age-specific sap flux densities are averaged values from 3 to 6 bamboo culms for each age category in one sunny day, where photosynthetic photon flux density larger than 35 mol m^–2^ day^–1^.

**FIGURE 5:**
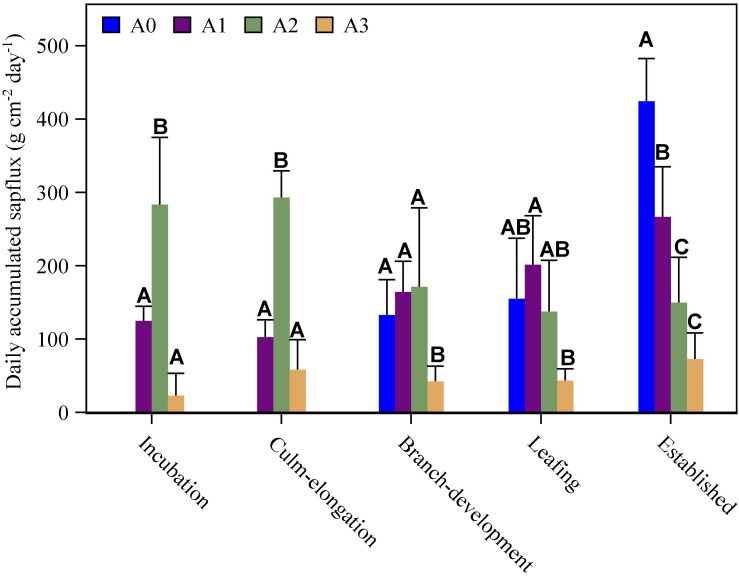
Comparison of daily accumulated sap flux density (g cm^–2^ day^–1^) of the three bamboo age categories in each spring phenological stages (incubation, culm-elongation, branch-development, leafing, established stages of newly sprouted culm). Different capital letters on top of error bars indicate significant differences of daily accumulated sap flux among the age categories in a specific stage.

Age had a significant impact on *J*_s_daily_ (*P* < 0.01; Table 2). *J*_s_ of A3 was always smaller than that of the other three age categories. *J*_s_daily_ of A2 was significantly higher than those of leaf-aging A1 and A3 in the incubation and culm-elongation stages of A0 (*P* < 0.01) while it was not different from A1 and A0 in branch-development and leafing stages of A0 when both have been flushing new leaves (*P* > 0.05). Furtherly, A2 was significantly surpassed by A1 and A0 in established stages of A0 when they have been expanding leaves (*P* < 0.01; Figures 3–5).

**TABLE 2:** ANOVA results about the effect of phenological stages and age on daily accumulated sap flux density.

**Hypothesis type**	**Source**	**DF**	***F* value**	***Pr* > *F***
I	Phenological stage	4	6.97	0.0002
	Age	3	27.70	<0.0001
III	Phenological stage	4	6.15	0.0004
	Age	3	28.80	<0.0001

**FIGURE 6:**
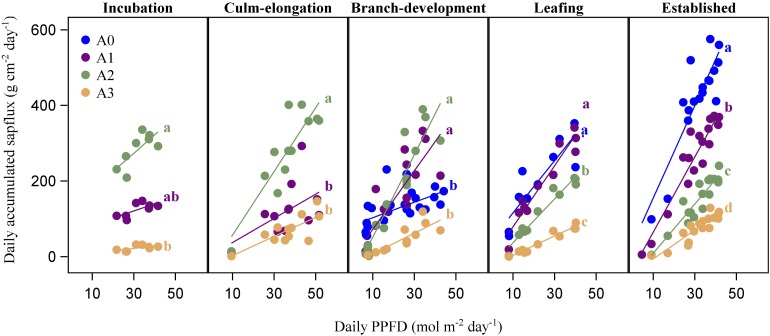
Relationships between daily photosynthetic photon flux density (PPFD; mol m^–2^ day^–1^) and daily accumulated sap flux density (g cm^–2^ day^–1^) for four age categories (**A0**, **A1**, **A2**, and **A3**) of Moso bamboo over five spring phenological stages (incubation, culm-elongation, branch-development, leafing, established stages of newly sprouted culm).

Except for *J*_s_daily_ of A3 in the incubation stage of A0, *J*_s_daily_ of the four ages in all the five stages was significantly and positively correlated with daily accumulated radiation (Appendix Table A1). Similar to the dynamic of *J*_s_ over the five periods, *J*_s_daily_ of A1, A2, and A3 responded to radiation gradually more, less, and constantly sensitive, respectively (Figure 6). In the culm-elongation stage of A0, A1 was less sensitive than A2, while A1 and A2 alternated their rankings after the branch-development stage of A0.

**FIGURE 7:**
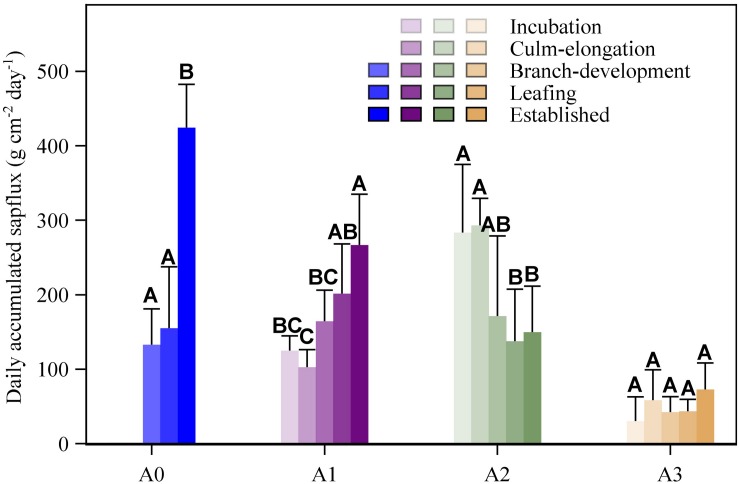
Comparison of daily accumulated sap flux density (g m^–2^ day^–1^) of the five phenological periods in each four bamboo age categories. Different capital letters on the top of the bar indicate significant differences in daily accumulated sap flux among five phenological periods for a specific age category.

Phenology had a significant impact on *J*_s_daily_ (*P* < 0.01; Table 2). The oldest culms – A3 kept no significant change over the whole spring phenological period (*P* > 0.05; Figure 7). A2, without renewing leaves, had significantly lower *J*_s_daily_ in the latter three stages (branch-development through leafing to established stages of A0) than in the earlier two stages (incubation and culm-elongation stages of A0). A1, which changed its leaves in spring, had significantly higher *J*_s_daily_ in the last stage (established stages of A0) when it had finished flushing and expanding new leaves than in the earlier four periods (incubation to leafing stages of A0). For the newly sprouted culms – A0, *J*_s_daily_ was almost two times higher in established stages than in branch-development and leafing stages (Figure 7).

### Daily Share of Stand Transpiration Among Age Categories

**FIGURE 8:**
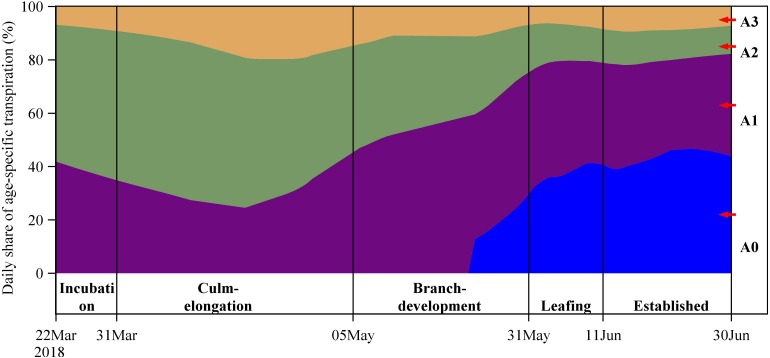
Daily share of age-specific stand transpiration among four age categories (**A0**, **A1**, **A2**, and **A3**) over five spring phenological period.

Daily share of stand transpiration among four age categories varied significantly over the five stages (Figure 8). Compared with A1 and A2, A3 always has the smallest share of stand transpiration. A2 has the largest share in the incubation and culm-elongation stages of A0, but A3 replaced A2 to have the largest share in brach-development and leafing stages of A0. In the last stage (established stage of A0), A0 became the leading one to occupy the largest share in daily stand transpiration. The four ages kept a nearly unchanged share of stand transpiration in the established stage when both freshly sprouted and established culms have already accomplished leaf-flushing and expanding.

## Discussion

During the experimental period (March to June 2018), the daily stand transpiration (the studied four age categories: newly sprouted, 1-, 2- and 3-year-old) varied from 0.29 to 2.17 mm d^–1^ and the mean value was 1.05 ± 0.47 mm d^–1^. Tentatively including culms with more than 3 years old and supposing them having the same water use as 3-year-old ones, the averaged daily stand transpiration would be 1.15 ± 0.52 mm d^–1^, which is equivalent to a yearly value of 420 mm. The studied stand transpiration fell into the range of those reported values (324–567 mm per year; Komatsu et al., 2010; Ichihashi et al., 2015; Zhao et al., 2017b).

Moso bamboo is supposed to have a decreasing water use with increasing ages due to the increased risk of xylem embolism, as they can not renew the vascular system by secondary growth as dicotyledonous trees do (Liese and Köhl, 2015). The negative age effect on water use of Moso bamboo was partially supported by our findings, in which the oldest 3-year-old culms (A3) had lower daily accumulated *J*_s_ than established ones of the other two age categories (A1, A2). Especially in the last stage when both newly sprouted culms and odd-year ones have new leaves, daily sap flow decreased significantly from the youngest culms (newly sprouted; A0) to the oldest ones (A3; Figures 3–7). The results confirmed two previous findings on Moso bamboo that the whole-culm transpiration decreased in order of juvenile, mature and senescence (Zhao et al., 2017b; Gu et al., 2019). Also in another study, despite age has an insignificant statistical effect, the older culms with more than 3-year-old have lower maximal *J*_s_ than 1- and 2-year-old culms (Tsuruta et al., 2016).

When we further explored the age effect coordinated with leaf phenology on water use of Moso bamboo, we found that over the whole spring phenological period, water use of different aged culms showed different dynamics, which broke the decreasing water use trend with increasing ages. One-year-old culms have lower *J*_s_ than 2-year-old ones before shedding leaves but exceed them after expanding leaves (*P* < 0.01; Figure 3). However, for the 3-year-old culms, which also experienced the same leaf-renewing procedure as the 1-year-old culms did, have an insignificant change in water use over the five phenological stages. As stomatal conductance is generally decreased with leaf aging (Davis and Mccree, 1978; Field and Mooney, 1983), newly expanded leaves are expected to have higher stomatal conductance and thus more water use. Therefore, although bamboo can adapt leaf conductance via regulating stomata to prevent damage in the conductive system (Yang et al., 2012, 2015), down-regulation of the renewed leaves may not fully offset xylem hydraulic dysfunction in the unrenewed vascular of aging culms due to embolism. Root pressure is another way to repair the embolized xylem (Cao et al., 2012; Yang et al., 2015), but the disadvantage of lacking secondary growth still seems to make culm embolism unavoidable over the years (Liese and Weiner, 1996).

In our study, the 2-year-old culms without renewing leaves have been decreasing their water use over the whole spring phenological period (Figures 3–7), which may be related to water transfer among the culms in the stand. On bamboos, previous studies have found translocation of nutrients (Li et al., 1998a, b; Song et al., 2016) and water (Fang et al., 2019) from established mature culms to those developing newly sprouted ones in growing seasons, which was thought to be crucial for the recruitment of new culms in “explosive growth” period (Song et al., 2016). When all the established mature culms were removed from a grove of *Gigantochloa apus*, water use of the left newly sprouted culms was reduced by nearly 80% (Fang et al., 2019). By applying the deuterium tracing method on some clumpy bamboo species, Dierick et al. (2010) and Mei et al. (2019) implied that water transfer might also exist among established clumpy culms. Compared to stand with lower culm density, stand with higher culm density had lower sap flux density and an earlier peak of stand transpiration (Ichihashi et al., 2015). The authors suggested the local microenvironment (e.g., radiation) may be a factor to induce the difference. However, higher stand culm density may mean more competition on water among culms. Thus each culm gets less water and cannot maintain high transpiration for a longer time than those in the stand with lower culm density.

These findings indicate that new leaf flushing benefits younger A1 and A0 more than those older culms (A2 and A3), i.e., dramatically increasing their transpiration response to radiation (Figure 6) and share of the stand transpiration (Figure 8). With the underground connected rhizome system, bamboo stand as an integration may balance its water use among different ages to support water use of freshly sprouted culms during their growth. Direct monitoring on rhizome showed the water support from established mother culms to the connected newly sprouted culms (Fang et al., 2019). Water transfer may act as a carrier to transfer carbohydrates (Song et al., 2016) and nutrients (Li et al., 1998b) from other culms to the newly sprouted culms. The freshly sprouted leaves (i.e., 1-year-old leaves) of bamboos were thought to have more active photosynthesis, which enables Moso bamboos to store carbohydrate for freshly sprouted culms in the next spring; at the meanwhile, the 2-year-old leaves may translocate their nutrients to other organs or freshly sprouted culms before they are shed (Li et al., 1998a, b). With shedding old and flushing new leaves, bamboo stands try to keep consecutive active productivity of carbohydrate and the following new recruitment. For the newly sprouted culms in a given year, they would benefit from an expanded foliar area and the corresponding leaf fresh weight (Huang et al., 2019a, b), i.e., by active photosynthesis and a re-allocation of nutrient and assimilated carbon into them. The advantages obtained from the spring leaf phenology may furtherly facilitate the development and survival of the newly sprouted culms in their first growing year.

## Conclusion

With 4-month (March to June) monitoring on sap flux densities in Moso bamboo culms of four age categories (newly sprouted, 1-, 2-, and 3-year-old culms), we partially confirmed the conventional negative age effect on water use by findings – lower water consumption in the 3-year-old culms than others over the whole experimental period and decreasing water use from youngest to oldest age categories at the end of the spring phenological period in June. However, the decreasing trends of water use with increasing ages seemed to be influenced by both the sprouting of new culms and leaf renewal of established ones in branch-development and leafing stages of the newly sprouted culms. During these two stages, water use of 2-year-old culms was reduced while that of the younger two age categories (A0 and A1) were increased. All these findings indicate that down-regulation of the renewed leaves may not fully offset xylem hydraulic dysfunction in the non-renewed vascular of aging culms, but leaf phenology could influence water redistribution in a bamboo stand to temporarily benefit younger A1 and A0 more than those older culms (A2 and A3), i.e., dramatically increasing their transpiration response to radiation and share of the stand transpiration. With shedding old and flushing new leaves, bamboo stands may keep consecutive active productivity of carbohydrate, which is important for the following new recruitment of culms. The findings can deepen our insight on the age and phenological effect on water use of Moso bamboo and thus provide some theoretical basis for bamboo management.

## Data Availability Statement

The datasets generated for this study are available on request to the corresponding author.

## Author Contributions

TM and XL contributed to the experimental design, field installations, data analysis, and wrote and revised the manuscript. DF contributed to the data analysis, figure-making, and revise the manuscript. GZ contributed to the experimental design and revision for the manuscript. CY contributed to the field installations. PL, YS, HD, FB, and DH contributed to revision for the manuscript.

## Conflict of Interest

The authors declare that the research was conducted in the absence of any commercial or financial relationships that could be construed as a potential conflict of interest.

## Footnotes

1 http://data.cma.cn

## Appendix

**TABLE A1:** Correlations between daily accumulated sapflux (three age categories) and PPFD in five phenological stages.

**Age**	**Phenological stages**				
	**Incubation**	**Culm-elongation**	**Branch-development**	**Leafing**	**Established**
A1	0.51*	0.45*	0.74**	0.93**	0.87**
A2	0.47*	0.74**	0.86**	0.96**	0.86**
A3	0.25	0.54**	0.65**	0.94**	0.81**

*The numbers are *R*^2^ of linear fit; * indicates significant correlation and ** very significant.*
